# Comparison of the efficacy and safety of Shanhuang Jiangzhi tablets and atorvastatin in the treatment of patients with hyperlipidaemia

**DOI:** 10.1186/s41043-023-00482-3

**Published:** 2023-12-14

**Authors:** GuoTong Sun, XiuWen Liang

**Affiliations:** 1 Suzhou Medical College of Soochow University, Soochow University, Suzhou, 215000 China; 2 Department of Cardiology, Hulunbuir Zhong Meng Hospital, No. 58 West Street, Hailar District, Hulunbuir, 021000 China; 3 Department of Cardiology, Shouguang Hospital of T.C.M, Weifang, 262700 China

**Keywords:** Traditional Chinese medicine, Tablets, Atorvastatin, Hyperlipidaemia

## Abstract

**Objectives:**

To compare the efficacy and safety of Shanhuang Jiangzhi tablets and atorvastatin in reducing blood lipid levels.

**Methods:**

Patients with hyperlipidaemia admitted to the cardiac centre between January 2019 and December 2020 were included in the study. A total of 1063 patients with hyperlipidaemia took either Shanhuang Jiangzhi tablets (*n* = 372) or atorvastatin (*n* = 691) and met the inclusion and exclusion criteria. Clinical data, including total cholesterol (TC), triglycerides (TG), low-density lipoprotein cholesterol (LDL-C) and high-density lipoprotein cholesterol, were retrospectively evaluated after propensity score matching (PSM) analysis. The adverse events were also recorded during the therapy process.

**Results:**

Following PSM analysis, both groups were well matched across all parameters. Compared with the baseline, Shanhuang Jiangzhi tablets had greater effects on TC, TG and LDL-C, and the difference was statistically significant (*p* < 0.001). Furthermore, the results showed that Shanhuang Jiangzhi tablets are similar to atorvastatin in reducing TC and LDL-C, and all *p*-values were > 0.05. However, the decrease of TG was greater in the Shanhuang Jiangzhi group (*p* < 0.001). Clinical adverse reactions of Shanhuang Jiangzhi tablets are rare and have no statistical significance compared with atorvastatin (*p* = 0.682).

**Conclusions:**

Shanhuang Jiangzhi tablets have a higher hypotriglyceridaemic performance than atorvastatin and an equivalent ability to lower TC and LDL-C. In addition, Shanhuang Jiangzhi tablets are a low-risk option for lowering blood lipids.

## Introduction

Hyperlipidaemia is a common and frequently occurring disease that is closely related to atherosclerotic cardiovascular and cerebrovascular diseases, such as coronary heart disease and stroke [1]. It can lead to damage to vascular endothelial function, increase oxidative stress and inflammatory response, promote the formation of foam cells, induce apoptosis or necrosis of endothelial cells and accelerate the pathological process of atherosclerosis [2]. Long-term intervention and control of blood lipids at appropriate levels, stabilising plaques and controlling the expression of vascular inflammatory factors are the basis for effectively preventing atherosclerosis and reducing the occurrence of cardiovascular and cerebrovascular events [3]. Therefore, the regulation of blood lipids is an important link in the control of atherosclerosis.

Statins are the mainstay treatment for hyperlipidaemia; however, their limitations include treatment resistance, intolerance due to adverse events and a lack of adherence, which contribute to poor outcomes [4]. Consequently, many patients require adjunct therapies, including niacin, bile acid sequestrants, fibric acids and ezetimibe, to effectively control hyperlipidaemia. In addition to Western medicine, some Chinese herbal medicines have demonstrated a lipid-lowering effect [5].

Shanhuang Jiangzhi tablets are a Chinese patent medicine that contains a mixture of Chinese herbal medicines, including Pueraria, Gynostemma, Polygonum multiflorum, Salvia, Alisma, turmeric, rhubarb, hawthorn, chuanxiong and cassia. Previous studies have shown that the treatment positively affects regulating blood lipids and can reduce atherosclerotic plaque [6]. Studies have shown that traditional Chinese medicines such as Salvia [7] and rhubarb [8, 9] are effective in improving haemodynamics, regulating adhesion factors in the blood and lowering lipid levels. Shanhuang Jiangzhi tablets contain the effective ingredients of various components of traditional Chinese medicine. According to the hospital's treatment experience, the therapeutic effect of Shanhuang Jiangzhi tablets is superior to that of any single traditional Chinese medicine. The tablet form also resolves the problem of difficulty in carrying and taking a decoction [10].

Considering the resistance and adverse reactions of Western medicine, exploring the treatment of hyperlipidaemia with traditional Chinese medicine is of great significance. This study compared the efficacy and safety of Shanhuang Jiangzhi tablets and atorvastatin in reducing blood lipid levels, thus to explore the mechanism of action of Shanhuang Jiangzhi tablets in treating hyperlipidaemia and provide more options for traditional Chinese medicine treatment of hyperlipidaemia.

## Participants and methods

### Study design, ethics approval and consent to participate

The clinical data of patients with hyperlipidaemia who received medication, including Shanhuang Jiangzhi tablets (Hulunbuir ZhongMeng Hospital, Hulunbuir, China) and atorvastatin (Pfizer Pharmaceutical Ltd., Dalian, China), at the cardiac centre between January 2019 and December 2020 were analysed. The composition of Shanhuang Jiangzhi tablets includes Pueraria, Gynostemma, Polygonum multiflorum, Salvia, Alisma, turmeric, rhubarb, hawthorn, chuanxiong and cassia. The content of the main component, puerarin, is 0.34 mg/tablet [11]. At present, no side effects have been observed. The current dosage is 2.4 g (eight tablets) per dose, twice a day. The study protocol was approved by the Ethics Committee of Hulunbuir ZhongMeng Hospital (Hulunbuir, China), who waived written informed consent due to the retrospective nature of the study. The PSM method was performed to compare the patients using Shanhuang Jiangzhi tablets with those using atorvastatin. The specific research methods were as follows. Before treatment, patient data were recorded for serological tests, including alanine aminotransferase (ALT), aspartate aminotransferase (AST), triglycerides (TG), total cholesterol (TC), high-density lipoprotein cholesterol (HDL-C) and low-density lipoprotein cholesterol (LDL-C), and these were used as the base value. After 2 months, the serological indexes were reviewed, and the medications adverse effects were recorded. The patients’ eating habits, lifestyle and other drug treatment schemes remained unchanged during the study. The types, manufacturers and dosage of drugs taken by each enrolled patient remained unchanged throughout the study.

## Research participants

The inclusion criteria were as follows: (1) patients who met the diagnostic criteria of TC ≥ 5.2 mmol/l, LDL-C ≥ 3.4 mmol/l and TG > 1.7 mmol/l) [12]; (2) patients who had no previous diagnosis of coronary heart disease, stroke, end-stage renal disease, cirrhosis, peripheral artery disease or heart failure; (3) patients who had no uncontrolled hypertension (blood pressure > 160/100 mmHg); (4) patients who had no uncontrolled diabetes mellitus (HbA1c > 7.5%); and (5) patients whose AST, ALT and serum creatine kinase levels were not more than twice the normal upper limit with no explainable cause, such as heavy exercise, muscle injury or massage. The exclusion criteria were as follows: (1) patients with acute cardiovascular and cerebrovascular diseases within the previous 6 months; (2) patients with abnormal metabolic function or complicated with severe hepatic and renal insufficiency and malignant tumour; (3) patients with mental illness, poor compliance or who could not follow the doctor's advice; (4) patients who had used other lipid-regulating drugs in the previous 2 weeks; and (5) patients who had an acute infection.

### Medication therapy

A total of 1063 patients met all the inclusion criteria and none of the exclusion criteria. The eligible patients were given either Shanhuang Jiangzhi tablets (*n* = 372) or atorvastatin (*n* = 691) based on the preference of the patient or the advice of the cardiologist. The dosage of Shanhuang Jiangzhi tablets was 2.4 g twice a day, and the dosage of atorvastatin was 20 mg daily. The duration of administration was 2 months.

### Data collection

The demographic information was obtained from the hospital information system and included age, gender, body mass index (BMI), smoking status, drinking status, hypertension and diabetes mellitus. A low (< 50%) ejection fraction (EF) was detected via routine echocardiography. The estimated glomerular filtration rate (eGFR) was calculated according to the cooperative equation of chronic kidney disease and epidemiology. The blood lipid levels of the two groups were compared before and after treatment. The detection method was as follows: 4 mL venous blood was taken on an empty stomach in the morning, and after standing for 20 min, it was centrifuged at a speed of 3,000 rpm for 10 min, and the obtained serum was kept in the refrigerator at − 20 ℃ for later use. The serum ALT, AST, TG, TC, HDL-C and LDL-C of patients were detected using a Cadic dry biochemical analyser (model: Cadic) in strict accordance with the instructions. All the information on adverse reactions that occurred during the treatment of A and B was recorded after reviewing the patient's medical records, temperature sheets, laboratory examination results and imaging examination results, while the evaluation was based on the evaluation criteria for adverse events (version 5.0), including the categories and grades of adverse reactions. The criteria for correlation evaluation were as follows: (1) whether there was a reasonable time relationship between medication and the occurrence of adverse reactions/events; (2) whether the reaction conformed to the known types of adverse reactions of the drug; (3) whether the reaction disappeared or decreased after stopping or reducing the dosage; (4) whether the same reaction/event occurred after using the suspect drug again; and (5) whether the reaction/event could be explained by the effect of combined medication, the progress of the patient's condition or the influence of other treatments. The evaluation results were divided into affirmative, probable, possible and possibly irrelevant, among which the adverse reactions that may have been irrelevant were judged as relevant [13].

### Statistics methods

All statistical analyses were performed using IBM SPSS version 26.0 software for Windows (SPSS Inc., Chicago, IL, USA). The PSM analysis was conducted using a multivariable logistic regression model based on demographic and biochemical information.

The propensity score value of each research object was obtained using the PSM method, wherein the drugs taken were the dependent variables, and the baseline data, such as age, sex, BMI, smoking history, drinking history, hypertension, diabetes mellitus, low EF, eGFR, ALT and AST, were the independent variables. Using PMS, the match tolerance was set to 0.01 in the logistic regression analysis. The Shanhuang Jiangzhi tablets group was matched at a 1:1 ratio to the atorvastatin group. This strategy resulted in 370 matched pairs across the two groups. The continuous measures were displayed as mean ± standard deviation, and the paired *t*-test was used to compare the differences. In addition, the categorical variables were expressed as counts and percentages (%), and the Chi-squared test or Fisher's exact probability method was performed in terms of the two groups. Unless otherwise specified, the test level was set at 0.05.

## Results

### Characteristics of participants

Table 1 shows the patient profiles before and after PSM. Clinical data identified 372 patients taking Shanhuang Jiangzhi tablets and 691 taking atorvastatin. The patients in the Shanhuang group were significantly older (*p* = 0.034) with lower TG (*p* = 0.026) before PSM than those in the atorvastatin group. However, the atorvastatin group contained a lower average LDL-C level (*p* = 0.001) and more smokers (*p* = 0.036) than the Shanhuang group. The patients were matched by PSM, where the match tolerance was set to 0.01, and 11 variables, including age, sex, BMI, smoking history, drinking history, hypertension, diabetes mellitus, low EF, eGFR, ALT and AST were matched at a ratio of 1:1. A total of 740 patients were included in the study, with 370 patients in the Shanhuang group and 370 patients in the atorvastatin group. There was no statistical difference in age, sex, BMI, smoking history, drinking history, tension, diabetes mellitus, low EF, eGFR, ALT and AST between the two groups (*p* > 0.05).

**Table 1:** Basic characteristics of subjects

Variables	Before propensity matching			After propensity matching		
	Shanhuang group *n* = 372	Atorvastatin group *n* = 691	*p*-value	Shanhuang group *n* = 370	Atorvastatin group *n* = 370	*p*-value
Age, y, mean ± SD	64.5 ± 12.6	62.8 ± 12.2	0.034	64.4 ± 12.6	64.1 ± 11.9	0.706
Males, n (%)	211(56.6)	406(58.8)	0.521	209(56.5)	206(55.7)	0.879
BMI, kg/m^2^, mean ± SD	25.6 ± 3.7	25.6 ± 2.9	0.857	25.5 ± 3.7	25.3 ± 3.5	0.334
Smoking status, n(%)	91(24.5)	211(30.5)	0.036	91(24.6)	93(25.1)	0.927
Drinking status, n(%)	100(26.9)	204(29.5)	0.363	98(26.5)	102(27.6)	0.804
Hypertension, n(%)	271(72.8)	506(73.2)	0.895	271(73.2)	266(71.9)	0.754
Diabetes mellitus, n(%)	118(31.7)	209(30.2)	0.619	117(31.6)	112(30.3)	0.743
Low EF(< 50%), n(%)	6(1.6)	8(1.2)	0.535	5(1.4)	3(0.8)	0.727
eGFR, ml/min/1.73m^2^, mean ± SD	91.8 ± 26.8	94.8 ± 27.5	0.089	91.8 ± 26.9	92.2 ± 27.0	0.821
ALT, U/L,mean ± SD	18.3 ± 9.3	18.0 ± 9.4	0.536	18.3 ± 9.3	18.6 ± 9.7	0.742
AST, U/L,mean ± SD	20.0 ± 6.6	20.8 ± 7.1	0.076	20.1 ± 6.6	20.1 ± 7.3	0.903
TC, mmol/L,mean ± SD	5.23 ± 0.89	5.15 ± 0.95	0.168	5.22 ± 0.87	5.22 ± 0.89	0.907
TG, mmol/L,mean ± SD	1.68 ± 1.27	1.88 ± 1.43	0.026	1.64 ± 1.04	1.64 ± 1.04	0.960
LDL-C, mmol/L,mean ± SD	3.55 ± 0.93	3.35 ± 0.94	0.001	3.56 ± 0.92	3.54 ± 0.94	0.725
HDL-C, mmol/L,mean ± SD	1.03 ± 0.23	1.04 ± 0.26	0.583	1.03 ± 0.23	1.02 ± 0.23	0.722

BMI: body mass index; EF: ejection fraction; eGFR:estimated glomerular filtration rate; ALT: alanine aminotransferase; AST:aspartate aminotransferase; TC:total cholesterol; TG:triglycerides; LDL-C: low-density lipoprotein cholesterol; HDL-C: high-density lipoprotein cholesterol

### Effect on serum lipid levels

The TC concentration decreased significantly (*p* < 0.01) during the 2-month treatment in the Shanhuang group. However, no significant difference was found in the 2-month intervention compared with the atorvastatin group (*p* = 0.466). The TG concentration in the Shanhuang group decreased significantly after 2 months of treatment (*p* < 0.001). The therapeutic effect in the Shanhuang group after 2 months was significantly greater than that in the atorvastatin group (*p* < 0.001). The LDL-C concentrations differed during the 2-month treatment in both the Shanhuang group and the atorvastatin group (*p* < 0.001), and no significant difference was noted between the groups (*p* = 0.169). Moreover, the HDL-C concentration did not differ significantly between the groups when measured before and after intervention (Table 2).

**Table 2:** The Serum Lipid Levels Before and After Intervention (mean ± SD)

Time	Variables	Shanhuang group	Atorvastatin group	*t*	*P*
Before treatment	TC	5.22 ± 0.87	5.22 ± 0.89	0.117	0.907
	TG	1.64 ± 1.04	1.64 ± 1.04	0.050	0.960
	LDL-C	3.56 ± 0.92	3.54 ± 0.94	0.353	0.725
	HDL-C	1.03 ± 0.23	1.02 ± 0.23	0.356	0.722
After treatment	TC	4.28 ± 0.56***	4.25 ± 0.56**	0.729	0.466
	TG	1.09 ± 0.52***	1.48 ± 0.72* ^###^	− 8.574	< 0.001
	LDL-C	2.76 ± 0.57***	2.82 ± 0.57**	− 1.376	0.169
	HDL-C	1.05 ± 0.23	1.02 ± 0.22	1.480	0.139

^*^: compare with before treatment *P* < 0.05; **: compare with before treatment *P*

< 0.01; ***: compare with before treatment *P* < 0.001; ^###^: compare with Shanhuang after treatment *P* < 0.001. TC:total cholesterol; TG:triglycerides; LDL-C: low-density lipoprotein cholesterol; HDL-C: high-density lipoprotein cholesterol

### Safety assessment

Clinical adverse effects were rare and comparable in the Shanhuang and atorvastatin groups (1.08% vs 0.54%, *p* = 0.682). There were four reported adverse effects in the atorvastatin group, including three patients with gastrointestinal discomfort and one patient with myalgias. Two patients in the Shanhuang group reported adverse reactions, both of which were gastrointestinal discomfort. No patients in the Shanhuang and atorvastatin groups had ALT or AST levels that exceeded three times the upper limit of normal.

## Discussion

The present study elicited three main findings. First, in the conventional treatment dose, Shanhuang Jiangzhi tablets were similar to atorvastatin in reducing TC and LDL-C. Second, compared with atorvastatin, Shanhuang Jiangzhi tablets can reduce the concentration of TG more effectively. Third, the clinical adverse reactions of Shanhuang Jiangzhi tablets are rare and have no statistical significance compared with atorvastatin.

The PSM statistical method was introduced by Rosenbaum and Rubin in the 1980s and is effective in dealing with non-randomised research data and control- or balance-confounding bias, making the research results close to those of randomised controlled research [7]. As a new method of balancing baseline data, PSM can integrate multiple confounding variables into one variable—namely tendency score—and effectively balance the distribution of confounding variables (also known as covariates) by balancing the tendency scores of two contrast groups, thus achieving the purpose of controlling confounding bias [9].

Statins reduce TC and LDL-C via the inhibition of 3-hydroxy-3-methylglutaryl-coenzyme A reductase (HMGR) [14] and are the mainstay of treatment for hyperlipidaemia [15]. Although statins are generally well tolerated, they are associated with numerous adverse effects, including hepatotoxicity [16], gastrointestinal events [17], musculoskeletal pain [18], respiratory infections [19] and headaches [20].

Many patients who take statins to treat hyperlipidaemia do not achieve optimal LDL-C goals, thus requiring additional treatment. Statin therapy can be complicated by adverse reactions (e.g. myalgias, elevated liver enzymes) and rare but life-threatening rhabdomyolysis. These issues provide an opportunity to consider the use of traditional Chinese medicine for patients who are non-adherent to statins, statin intolerant or statin resistant.

As an effective supplementary and alternative treatment, traditional Chinese medicine has attracted increasing attention. Chinese medicinal herbs are regarded as a rich source of natural drug development. Shanhuang Jiangzhi tablets are composed of 10 traditional Chinese medicines. Pueraria is one of the commonly used herbs for the treatment of hyperlipidaemia. Network pharmacology has found that the key active ingredient in the cholesterol-lowering effect of puerarin (Pueraria active extract) is β-sitosterol [21]. Liu et al. found that the increase of TC, TG and LDL-C induced by lead was effectively suppressed by puerarin. The HDL-C level in the lead treatment of rats was also increased by puerarin. Western blot analysis showed that puerarin remarkably inhibited hyperlipidaemia by regulating the expression of cholesterol 7a-hydroxylase (CYP7A1), HMGR and low-density lipoprotein receptors in the liver of lead-treated rats [22].

Gynostemma is widely used for the treatment of diseases such as hyperlipidaemia, fatty liver and obesity in China. Using H nuclear magnetic resonance spectra (^1^H-NMR)-based metabolomics, Wang et al. elucidated the therapeutic mechanisms of Gynostemma. It exerts its antihyperlipidaemic effect by elevating the level of phosphatidylcholine and decreasing the level of trimethylamine N-oxide [23].

Likewise, Polygonum multiflorum is widely used in the prevention and treatment of hyperlipidaemia in traditional Chinese medicine. The LDL-C, TC and TG of hyperlipidaemia rats treated with Polygonum multiflorum were significantly decreased. The key enzymes involved in lipid metabolism, HMGR, fatty acid synthase and acetyl-CoA carboxylase (ACC) in plasma were generally reduced after oral administration, which was consistent with the transcription levels of their target genes [24].

Based on lipidomic technology and network pharmacology analysis, it is thought that Salvia miltiorrhiza prevents and treats hyperlipidaemia through salvianolic acid A [25]. Alisma targeting the FKBP38/mTOR/SREBPs pathway improves hyperlipidaemia [26]. Turmeric is mainly involved in arachidonic acid metabolism, steroid hormone biosynthesis and the peroxisome proliferator-activated receptors signalling pathway to reduce the plasma TC, TG and LDL-C levels of high-fat diet-fed mice [27]. Hawthorn significantly reduces levels of TC, TG and LDL-C, with n-butanol and ethyl acetate having the highest efficacy [28, 29]. Cassia can treat hyperlipidaemic animals with elevated TC, TG, LDL-C and very low-density lipoprotein cholesterol. Hyperlipidaemia alters the protein and messenger ribonucleic acid expression levels of the key genes (sterol regulatory element-binding protein-1c, ACC1, sterol regulatory element-binding protein-2, HMGR, 3-hydroxy-3-methylglutaryl-CoA synthase, CYP7A1 and ATP-binding cassette transporter A1) in lipid metabolism and the treatment with cassia reverts these levels to those observed with atorvastatin-treated hyperlipidaemic animals [30].

In terms of safety indicators, neither the atorvastatin group nor the Shanhuang group had the risk of elevated liver enzymes. Of the 370 patients on atorvastatin, one developed muscle pain and laboratory indicators suggestive of elevated creatine kinase. However, there was no adverse reaction of muscle pain in the Shanhuang group. Only two cases had symptoms of diarrhoea, and considering the effect of rhubarb on increasing bowel movements and promoting defecation, no special treatment was given.

This study was based on a hospital database and had several limitations. First, there may have been selection bias due to the choice of drugs based on patient or cardiologist preferences. There are inherent differences in the patients who are selected for treatment with atorvastatin versus Shanhuang in routine clinical practice. Second, PSM was used to balance the potential differences between the two study groups; however, some parameters were not considered and may have confounded the study results, which is an inherent limitation of retrospective studies. Third, the sample size of 740 patients is relatively small. Moreover, the period for following up the included trial is short. Finally, the research outcomes might also be influenced by the patients’ personal diet and lifestyle, which is difficult to control.

## Conclusions

This study demonstrates that Shanhuang Jiangzhi tablets have a higher hypotriglyceridaemic performance than atorvastatin and an equivalent efficacy in lowering TC and LDL-C. In addition, Shanhuang Jiangzhi tablets are a low-risk option for lowering blood lipids.

## Data Availability

All data generated or analysed during this study are included in this published article.
